# The Hidden Link in the Kinematic Chain: The Influence of the Tossing arm on Head and Serve Kinematics in Tennis

**DOI:** 10.1002/ejsc.70022

**Published:** 2025-07-25

**Authors:** Oliver Newton, Olivier Girard, Aaron Chin, Machar Reid

**Affiliations:** ^1^ School of Human Sciences (Exercise and Sports Science) The University of Western Australia Perth Australia

**Keywords:** ball toss, coaching, cue, service technique, task constraint

## Abstract

The control and placement of the ball toss are critical features of the tennis serve. Whereas much attention has been given to the ball's trajectory, the motion of the tossing arm has been overlooked. This study aimed to describe the kinematics of the tossing arm and its impact on full‐body kinematics and serve performance. Ten high‐performance male tennis players were studied using a 10‐camera, 250 Hz VICON T‐series motion analysis system capturing full‐body, racquet, and ball kinematics. Each participant executed ten maximum effort first serves with instructions to (i) *serve normally*, (ii) *adduct the tossing arm early* and (iii) *adduct the tossing arm late*. While head kinematics remained unaffected by the serve condition, the ball toss was higher and further forward in both altered conditions, increasing serve duration (*p* < 0.001). Players compensated by extending the preparation phase's proportion of the serve by 4.3% in both conditions. Serve trajectory was ∼3° steeper downwards (*p* < 0.001) in the early condition and ∼1° flatter (*p* = 0.020) in the late condition than participants' normal serve. In summary, alterations to the tossing arm elicited the expected changes in the serve's post‐impact ball trajectory, but coaches must monitor several unintended consequences.

## Summary

- Compared to the player's normal serves, adducting the tossing arm earlier resulted in a steeper downward ball trajectory, whereas adducting the tossing arm later resulted in a flatter ball trajectory, hindering its accuracy.
- In both tossing arm conditions, the ball toss was higher and further forward; however, it had no significant impact on the position of the head.
- Altering the timing of tossing arm adduction impacted the serve's temporality, where players would extend the preparation phase in proportion to the entire service motion

## 1 Introduction

The serve is widely regarded as the most important shot in tennis, and its biomechanical determinants have been extensively researched (Elliott and Wood 1983; Reid et al. 2011; Giblin et al. 2017). It stands out as the only closed skill in the sport, giving the server a unique advantage in setting the tone of the point from the start (Elliott and Wood 1983; Reid et al. 2010). The coordination of both upper and lower body segments, beginning with knee and hip joint extension, followed by trunk rotation, racquet arm extension and the resulting racquet rotation through impact, forms the basis of the serve's intricate kinematic chain (Elliott and Kilderry 1983; Roetert and Kovacs 2019). The ball toss initiates this chain, and unlike any other shot in tennis, a player can elect not to hit the ball during the service motion, highlighting its importance (Fett et al. 2021). Despite this, no empirical work has specifically examined the influence of the tossing arm on the serve's full‐body kinematics, including head position.

Servers attempt to deceive their opponents by varying spins and trajectories (Vacek et al. 2023; Whiteside et al. 2014, 12 july ‐ 16 july). Interviews with eight ATP male professionals concluded that returners primarily use the ball toss as a kinematic cue to anticipate a serve (Vernon et al. 2018). Accordingly, Reid et al. (2011) examined the consistency of the ball toss when serving to different locations within the service box. They reported significant differences in the lateral displacement of the ball at impact and its zenith between serves directed at the body and ‘T’. This implies that the lateral displacement of the toss might act as a directional anticipatory cue for returners. Carboch and Süss (2015) additionally revealed that the contact point for the kick serve was significantly lower (5.7 cm) than for the slice serve when targeting the same part of the box. Both studies indicate that returners can gather predictive information from the toss, although it is unclear how discernible these differences are for returners standing ∼25 m away. Nevertheless, given that the toss is largely a product of the motion of the tossing arm, the lack of scrutiny applied to tossing arm kinematics is surprising.

The tossing arm's adduction in the service action facilitates the optimisation of the trunk and hitting arm's stretch‐shortening cycle, an important part of the kinematic chain in many overhand sports (Seroyer et al. 2010; Kovacs and Ellenbecker 2011). It is also thought to be linked to the head's orientation and gaze during the forward swing. Whereas considerable discussion has centred on the head's role in groundstrokes (Lafont 2008) and other interceptive sports (Shinkai et al. 2022; Mann et al. 2013), its influence on the serve has been largely overlooked. Common coaching interventions that address the tossing arm manipulate the timing of its adduction to elicit changes in the head's position and serve performance. However, these cues and the relationship between the head's position and serve trajectory are often devised through trial and error, lacking supporting empirical evidence (Reid et al. 2013). Williams and Petersen (2000) described a prevalent coaching cue where players are encouraged to delay the adduction of the tossing arm. This cue is believed to help players maintain a higher head position and flatten the service trajectory to avoid net faults (Williams and Petersen 2000). Conversely, when serves land long due to an overly flat trajectory, players are instructed to adduct their tossing arm earlier to steepen it. However, the efficacy of these instructions is unknown and, like other previously investigated interventions (Reid et al. 2013), may inadvertently impair serve performance.

Accordingly, this study aimed to comprehensively examine the influence of altering the timing of tossing arm adduction on full‐body kinematics and tennis serve performance. Compared to a normal serve, early adduction of the tossing arm was hypothesised to increase head and trunk flexion at impact, steepen the ball trajectory, lower the impact point and shorten service duration. Conversely, late adduction was expected to decrease head and trunk flexion at impact, flatten ball trajectory, raise the impact point and lengthen the service action. Serve velocity was anticipated to be unaffected, whereas serve accuracy was expected to decline with any modification in tossing arm adduction.

## 2 Methods

### 2.1 Participants

Ten right‐handed, high‐performance (Universal Tennis Ranking: 10.7 ± 1.3), male tennis players (mean ± SD age: 20.3 ± 2.4 years, body mass: 77.3 ± 7.9 kg and height: 186.7 ± 6.8 cm) participated in this study. All participants were uninjured and naturally served with the foot‐up and forward ball toss technique to minimise confounding variables (Martin 2015; Knudson and Bahamonde 2001; Elliott and Wood 1983).

Selection of the appropriate sample size was conducted through a power analysis using customised computer software (GPOWER Version 3.1, Department of Psychology, Bonn University, Bonn Germany). Using the following input parameters—effect size = 0.69, power of 0.80 and alpha of 0.05—determined by previous research investigating the effects of leg contribution on serve velocity (Girard et al. 2007), the recommended sample size was 11. However, due to the limited accessibility of high‐performance players who meet the inclusion criteria, 10 participants were used.

Ethical approval was granted by the Human Research Ethics Committee (ROAP2023/ET000451), and participants provided signed informed consent before inclusion in the study.

### 2.2 Experimental Protocol

Players were required to hit 10 serves under three different conditions: (i) normal serves with no external cue (NORMAL), (ii) early adduction of the tossing arm (EARLY) and (iii) later adduction of the tossing arm (LATE). Importantly, these instructions are common in tennis coaching and typically do not specify a particular time interval. Players are left to self‐select what ‘adduct early’ and ‘adduct later’ mean to them, which was the approach adopted in this study to maintain ecological validity.

A familiarisation session was conducted 1 week before the experiment. During this session, participants were affixed with a full‐body set of markers and asked to practice 10 serves in each condition. This ensured that the players had prior experience executing serves in all conditions.

On the day of testing, after completing their individual warm‐up routines, players were instructed to execute maximum effort first serves (flat with minimal spin) aiming for a 2 × 1‐m target area bordering the ‘T’ of the left service box (Reid et al. 2010; Whiteside et al. 2014). Each player started with the 10 NORMAL serves; then, to limit potential order effects, the two tossing arm conditions were counterbalanced among the participants. A 2‐min rest period between blocks of 10 serves was provided to minimise fatigue effects.

### 2.3 Subject and System Preparation

A full‐sized tennis court was demarcated with tape inside the UWA School of Human Sciences Biomechanics lab. A total of 54 retroreflective markers (12 mm in diameter) were affixed to each participant's body in accordance with the UWA full‐body marker set protocol (Besier et al. 2003; Campbell et al. 2009). Due to the novelty of quantifying head kinematics and to ensure consistency in marker placement, two additional markers were placed on the zygomatic arches. Five markers were attached around the frame of the tennis racquet, and three hemispherical markers composed of ultralight foam (< 0.07 g) were attached to the ball to track their respective coordinate systems. Players used their own racquets and were given 10 min to complete their usual match day warm‐up routine. Serving indoors also controlled for constraining factors such as the wind, sun position and temperature.

A 10‐camera VICON T‐series motion capture system (Vicon Motion Systems, Oxford, UK), collecting at 250 Hz, tracked three‐dimensional marker trajectories within an 84 m^3^ capture volume. The global origin was calibrated to coincide with the intersection of the centreline and the baseline, and in this coordinate system, the *x*‐axis pointed to the right, the *y*‐axis pointed towards the net and the *z*‐axis pointed upwards.

### 2.4 Data Treatment

For each participant, three serves out of the block of 10 were selected to best represent the provided cue. From the 10 NORMAL serves, only the first three that successfully landed in the target box were considered successful. From the 10 serves executed under both tossing arm conditions, those with the shortest and longest delays in adducting the tossing arms were selected for further analysis. These trials were selected regardless of the service outcome. This selection approach was mainly based on two considerations: (1) the limited number of serves that successfully landed in the target area for both tossing arm conditions and (2) the assumption that the most exaggerated delays would provide the most noticeable contrast to normal serve kinematics, thereby potentially highlighting the intended effect of both interventions.

To address gaps in the raw marker trajectories, the cubic spline interpolation was employed (Reid et al. 2013). A low‐pass Butterworth filter with a mean squared error of 25 Hz, as determined by a residual analysis, was then applied to all data. Finally, the UWA full‐body, racquet and ball models were applied to the filtered data to produce the kinematic and temporal variables of interest (Besier et al. 2003; Campbell et al. 2009; Whiteside 2012).

### 2.5 Determination of Events, Phases and Variables

Four key events were identified for each serve: *ball release* (*BR*), *trophy position* (*TP*)—the racquet high point during the preparatory action for the stroke (Whiteside et al. 2013), *racquet low point* (*RLP*)—the lowest displacement of the racquet occurring after trophy position and *impact*—occurring 0.04 s before racquet and ball impact, due to the 250 Hz sampling frequency. Ball impact was defined as the frame with the smallest absolute distance between the ball markers and the racquet frame.

These events, defined based on the racquet's position, allowed for the categorisation of three distinct phases: the *preparation* phase, between *BR* and *TP*, the *propulsion* phase, between *TP* and *RLP*, and the *forward swing* phase, between the *RLP* and *impact*.

The following kinematics, as informed by previous research (Reid et al. 2013; Whiteside et al. 2013; Fleisig et al. 2003), were assessed to test the study's hypotheses:

– Outcome measures: Service accuracy was categorised based on the following landing locations: (i) in the 2 × 1‐m target area, (ii) in the service box but missing the target, (iii) hitting the net and (iv) landing long of the service box. Other measures included trajectory (the angle between the ball at *impact* and five frames post‐*impact*, measured in the global sagittal plane) and peak racquet head velocity. An external experimenter, positioned courtside, evaluated the landing location.
– Head kinematics: Extension angle of the head relative to the global axis was measured at the *TP*, *RLP* and *impact*.
– Upper body kinematics: The racquet arm's wrist and the thorax angle relative to the global axis and the adduction angle of the tossing arm, and additionally, the peak shoulder alignment rotation in the *preparation* phase, the range of shoulder alignment rotation in the *forward swing* and the peak shoulder‐over‐shoulder angular velocity in the *forward swing*, were also included.
– Lower body kinematics: peak knee flexion angle and peak hip vertical velocity of both legs
– Toss kinematics: vertical and forward ball positions at *impact* and the height of the ball zenith
– Temporal characteristics: absolute and relative time of the key events in relation to the service action

### 2.6 Statistical Analysis

One‐way analyses of variance, with accompanying Bonferroni post hoc analyses, were used to test the study's hypotheses. For all tests, differences were assessed for significance where *p* < 0.05. All analyses were performed on SPSS version 27.0 (SPSS Inc., Chicago, IL.)

## 3 Results

### 3.1 Serve Outcome and Ball Toss Kinematics

When the adduction of the tossing arm was delayed, the service trajectory significantly flattened (Table 1). Post‐*impact* trajectories were ∼3° steeper for EARLY and ∼1° flatter for LATE than NORMAL. This change in trajectory affected serve accuracy, with 90% of serves hitting the net when the arm was adducted early, and 63% going long when the arm was adducted late. Although the height of the ball toss in NORMAL was lower than LATE (*p* = 0.045), impact height remained consistent regardless of tossing arm adduction. However, the forward position of the ball at *impact* was significantly farther into the court in the EARLY (67.01 ± 16.60 cm) and LATE (69.87 ± 16.45 cm) conditions than NORMAL (53.46 ± 15.75 cm; *p* < 0.001). Racquet velocity at *impact* did not differ among the conditions.

**TABLE 1:** Comparison of the outcome measures of serve performance and spatial characteristics of the toss among the NORMAL, EARLY and LATE conditions.

Variable	Unit	NORMAL		EARLY		LATE		ANOVA		Post hoc		
		Mean	S.D.	Mean	S.D.	Mean	S.D.	*F*	*p*	N versus.EARLY	N versus. LATE	EARLY versus. LATE
Service performance												
Racquet velocity at impact	km/h	157.42	8.00	156.16	6.83	153.93	8.53	1.526	0.223			
Service trajectory	deg	6.87	1.04	9.80	2.25	5.67	1.49	48.577	< 0.001*	*p* < 0.001	*p* = 0.020	*p* < 0.001
Landing location		**Percentage**		**Percentage**		**Percentage**						
In (the target area)	%	87		3		17						
Good (landing in the service box)	%	13		7		10						
Net	%	—		90		10						
Long	%	—		—		63						
Ball toss kinematics												
Ball position at BZ: z	cm	347.66	24.01	356.59	10.62	362.49	21.81	3.119	0.049*		*p* = 0.045	
Ball position at impact: z	cm	291.46	9.94	292.74	10.62	292.63	10.63	0.140	0.869			
Ball position at impact: y	cm	53.46	15.75	67.01	16.60	69.87	16.45	8.709	< 0.001*	*p* = 0.005	*p* < 0.001	

Abbreviations: EARLY = ‘early adduction of the tossing arm’ condition; LATE = ‘late adduction of the tossing arm’ condition; NORMAL = ‘normal serve’ condition.

*Significant at < 0.05 level.

### 3.2 Temporal Characteristics of the Serve

Manipulating the absolute timing of tossing arm adduction (∼12% earlier in EARLY and ∼10% later in LATE) led to significant changes in service rhythm (Table 2). Players achieved the *TP* later in EARLY and LATE (*p* = 0.032 and *p* < 0.001, respectively). In addition, the time taken to reach the *RLP* (*p* = 0.002) and *impact* (*p* < 0.001) was longer in the LATE than NORMAL. Players spent a longer duration of time in the *preparation* phase as a proportion of the total serve in both EARLY (68.8 ± 4.7%) and LATE (68.8 ± 4.0%) than NORMAL (64.5 ± 8.4%). Conversely, the time players spent in the *propulsion* phase was significantly shorter (3.7%; *p* = 0.034) when the tossing arm adduction was delayed. These changes in serve timing enabled the relative duration of the *forward swing* phase to remain consistent across all three conditions.

**TABLE 2:** Comparison of the temporal characteristics of the service action among the NORMAL, EARLY and LATE conditions.

Variable	Unit	NORMAL		EARLY		LATE		ANOVA		Post hoc		
		Mean	S.D.	Mean	S.D.	Mean	S.D.	*F*	*p*	N versus. EARLY	N versus. LATE	EARLY versus. LATE
Key event timing:												
Timing of trophy position	s	0.62	0.11	0.69	0.09	0.72	0.08	8.730	< 0.001*	*p* = 0.032	*p* < 0.001	
Timing of racquet low point	s	0.84	0.10	0.88	0.09	0.92	0.08	6.123	0.003*		*p* = 0.002	
Timing of impact and total serve duration	s	0.97	0.10	1.00	0.08	1.05	0.08	7.684	< 0.001*		*p* < 0.001	
Phase timing:												
Preparation as a proportion of total serve	%	64.5	8.4	68.8	4.7	68.8	4.0	5.127	0.008*	*p* = 0.032	*p* < 0.001	
Propulsion as a proportion of total serve	%	22.3	8.0	18.8	4.3	18.6	3.7	4.167	0.019*		*p* = 0.034	
Forward swing as a proportion of total serve	%	13.2	2.1	12.4	1.8	12.6	1.6	1.446	0.241			
Toss to tossing arm adduction as a proportion of total serve	%	64.7	7.7	52.7	10.6	74.9	6.5	51.487	< 0.001*	*p* < 0.001	*p* < 0.001	*p* < 0.001

Abbreviations: EARLY = ‘early adduction of the tossing arm’ condition; LATE = ‘late adduction of the tossing arm’ condition; NORMAL = ‘normal serve’ condition.

*Significant at < 0.05 level.

### 3.3 Lower Limb, Trunk and Upper Limb Kinematics

**FIGURE 1:**
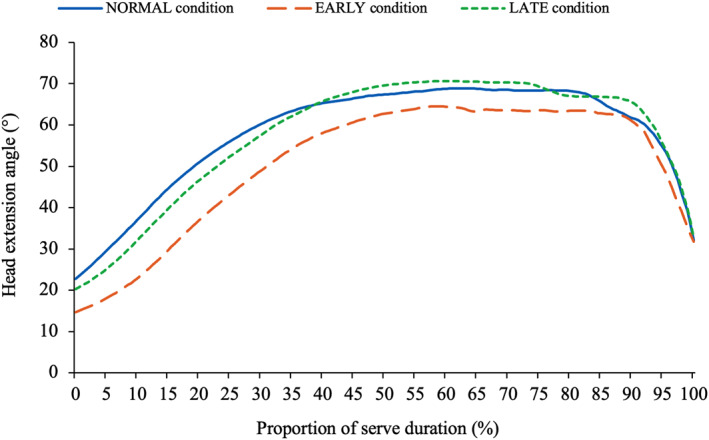
The head extension angle in the NORMAL, EARLY and LATE conditions *Significant at < 0.05 level. EARLY = ‘Early adduction of the tossing arm’ condition; LATE = ‘Late adduction of the tossing arm’ condition; NORMAL = ‘Normal serve’ condition.

**FIGURE 2:**
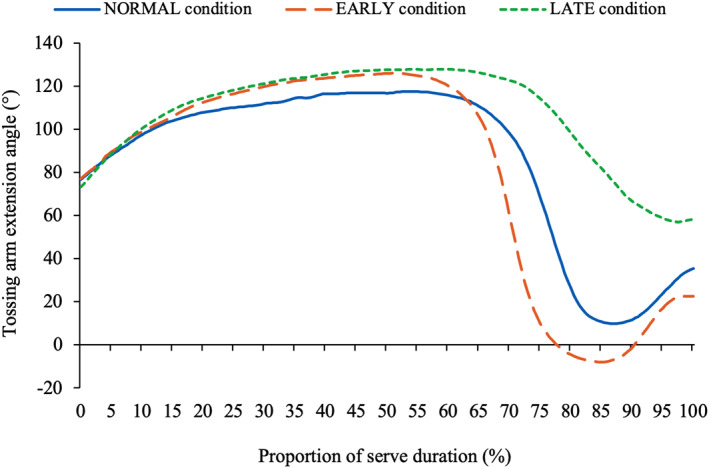
The tossing arm adduction angle in the NORMAL, EARLY and LATE conditions *Significant at < 0.05 level. EARLY = ‘Early adduction of the tossing arm’ condition; LATE = ‘Late adduction of the tossing arm’ condition; NORMAL = ‘Normal serve’ condition.

**FIGURE 3:**
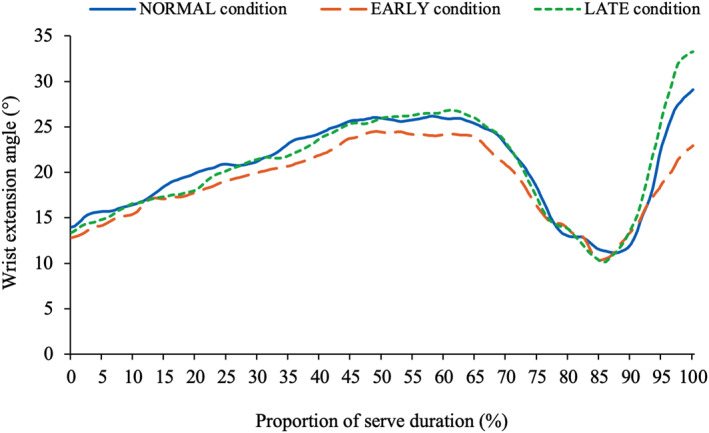
The wrist extension angle of the racquet arm in the NORMAL, EARLY and LATE conditions *Significant at < 0.05 level. EARLY = ‘Early adduction of the tossing arm’ condition; LATE = ‘Late adduction of the tossing arm’ condition; NORMAL = ‘Normal serve’ condition.

The kinematics of the head remained relatively consistent, independent of tossing arm action (Table 3). Figure 1 displays the head's extension angle across all conditions throughout the entire service action, with differences observed only in the extension angle of the head in the *TP* (*p* = 0.034), primarily between EARLY and LATE (*p* = 0.040). Conversely, racquet arm kinematics were strongly influenced by changes in tossing arm adduction timing. In EARLY, the tossing arm adduction angle at *impact* was 13° lower, whereas in LATE, it was 22.6° higher. The kinematics of the tossing arm also differed considerably between the three conditions (Figure 2). In EARLY, the tossing arm's adduction angle dropped lower than in both other conditions (NORMAL: 9.8°; EARLY: −7.9°; LATE: 56.8°) and at a faster rate. However, as a result, during the *forward swing* phase, the tossing arm was forced to move to a more familiarly extended position at *impact*. This pattern was also observed in NORMAL, where the tossing arm began extending at around 70%–75% of the swing duration and continued through to *impact*. However, the LATE condition did not exhibit a similar extension in the tossing arm prior to *impact*. The racquet arm was more extended at *impact* in NORMAL than EARLY and LATE (166.1 ± 5.0° vs. 156.8 ± 4.4° and 156.2 ± 4.2°), and the racquet arm's wrist was more flexed when the tossing arm dropped earlier. No differences in the wrist angle were observed until 90%–95% through the service action (Figure 3). Players then extended the wrist further in the LATE condition, and less in EARLY, manipulating the angle of the racquet moments before *impact*.

**TABLE 3:** Comparison of full‐body kinematics of the service action among the NORMAL, EARLY and LATE conditions.

Variables	Time reference	Unit	NORMAL		EARLY		LATE		ANOVA		Post hoc		
			Mean	S.D.	Mean	S.D.	Mean	S.D.	*F*	*p*	N versus. EARLY	N versus. LATE	EARLY versus. LATE
Head													
Head extension angle	TP	deg	68.8	10.7	63.6	10.5	70.3	9.6	3.516	0.034*			*p* = 0.040
Head extension angle	RLP	deg	63.9	9.9	62.4	10.4	66.6	10.4	1.270	0.286			
Head extension angle	Impact	deg	32.1	9.1	31.8	8.7	32.6	8.8	0.072	0.930			
Arms													
Tossing arm adduction angle	Impact	deg	35.5	19.3	22.5	15.8	58.1	31.3	18.298	< 0.001*	*p* = 0.031	*p* < 0.001	*p* < 0.001
Racquet arm extension angle	Impact	deg	166.1	5.0	156.8	4.4	156.2	4.2	43.94	< 0.001*	*p* < 0.001	*p* < 0.001	
Racquet arm wrist extension angle	Impact	deg	29.1	5.9	22.9	5.2	33.3	6.3	24.78	< 0.001*	*p* < 0.001	*p* = 0.018	*p* < 0.001
Trunk													
Thorax extension angle	Impact	deg	23.6	6.2	27.0	5.4	20.1	6.0	9.822	< 0.001*			*p* < 0.001
Peak shoulder alignment rotation in the preparation phase		deg	35.4	13.8	42.4	12.4	31.3	12.7	5.642	0.005*			*p* = 0.004
Range of shoulder alignment rotation in the forward swing		deg	86.1	13.4	93.4	14.8	79.7	12.7	7.569	< 0.001*			*p* < 0.001
Peak shoulder‐over‐shoulder angular velocity	Impact	deg/s	643.2	67.2	629.5	48.7	622.5	58.0	0.974	0.382			
Lower body													
Peak front hip vertical velocity		m/s	1.41	0.42	1.46	0.49	1.50	0.41	0.267	0.767			
Peak back hip vertical velocity		m/s	2.10	0.39	2.11	0.49	2.17	0.38	0.231	0.794			
Peak front knee flexion angle		deg	75.3	10.0	73.9	11.0	75.5	8.8	0.219	0.804			
Peak back knee flexion angle		deg	88.2	12.0	86.8	14.1	89.2	12.1	0.277	0.759			

Abbreviations: EARLY = ‘early adduction of the tossing arm’ condition; LATE = ‘late adduction of the tossing arm’ condition; NORMAL = ‘normal serve’ condition.

*Significant at < 0.05 level.

Multiple differences existed in the trunk's kinematics, primarily between EARLY and LATE. The extension angle of the thorax (*p* < 0.001), peak shoulder alignment rotation in the *preparation* phase (*p* = 0.004) and the range of shoulder alignment rotation in the *forward swing* (*p* < 0.001) were significantly higher in EARLY than NORMAL. In contrast, the peak shoulder‐over‐shoulder angular velocity, as well as all lower limb kinematics, did not differ between conditions.

## 4 Discussion

Manipulating the timing of tossing arm adduction significantly impacted serve performance. Instructing participants to adduct their tossing arms early and late resulted in a trajectory ∼3° steeper downwards and ∼1° flatter, respectively, than their normal serve. While this change aligned with the coaching cue's intention of altering service trajectory, it unexpectedly impacted other kinematic and temporal aspects of the serve. Overall, coaches need to be mindful of how such cues may elicit the desired performance change while also impacting other critical skill components.

### 4.1 Serve Outcome and Ball Toss Kinematics

As players had to alter their natural service technique, they notably elevated the ball toss height and contacted the ball farther out in front (Table 1). Even though the average ball toss height increased in both conditions, the difference was significant only between LATE and NORMAL (4.27% higher; *p* = 0.045). A similar perturbation in toss height was observed in Reid et al. (2010), when players were asked to rehearse their toss in isolation of ball impact. Surprisingly, contact with the ball occurred significantly farther into the court in both EARLY and LATE, contrary to our hypothesis. Variability in ball toss can be attributed to the constraints‐based perspective of dynamic systems theory (Davids et al. 2006), indicating that behaviours are influenced by the adaptive interaction between person, environment and task. Adding a task constraint compelled players to modify their familiar technique, reflecting the flexible and adaptive motor system (Davids et al. 2001). The heightened toss directly extended serve duration (Table 2), likely due to the motor system requiring more time to process unfamiliar perceptual information and accommodate a larger margin for timing error (Davids et al. 2001). Considering that a consistent ball toss is critical for effective serve performance (Fett et al. 2021), variability between conditions likely contributed to the observed differences in serve trajectory and overall execution.

### 4.2 Temporal Characteristics of the Serve

The rhythm of the serve is critical in developing consistency and optimising effectiveness (Reid january 7, 2014). As previously discussed, the increased vertical ball zenith corresponded to a prolonged total serve duration (Table 2). To pinpoint where players slowed down their service actions, the serve was divided into three phases: *preparation*, *propulsion* and *follow‐through* (Whiteside et al. 2013). Compared to NORMAL, the *preparation phase* was extended by 4.4% in EARLY and LATE (Table 2), which concurred with our hypothesis. This difference could theoretically result from players gradually delaying their racquet takeback or maintaining the *TP* until initiating the *propulsion* phase.

The trophy position, marked as the racquet high point during the preparatory action for the stroke (Whiteside et al. 2013), is coordinated with tossing arm movement to produce a smooth and efficient service action. If players pause at the *TP*, increasing the duration of the amortisation phase (the time spent in the eccentric pre‐stretch of a muscle), the muscle's ability to generate force concentrically can be reduced (Elliott 2006). Therefore, a decrease in racquet head velocity (Elliott et al. 1999) and shoulder‐over‐shoulder angular velocity (Bahamonde 2000) might be expected. However, Tables 1 and 3 indicate that neither variable was affected in this way. While beginners may be prone to pause at the TP, waiting for the ball to drop into their hitting zone (Whiteside et al. 2013), the more experienced players in the current study appeared to be able to synchronise the height of the ball toss with the timing of their takeback to preserve these select upper limb and racquet kinematics. Coaches must nevertheless be aware of how such task constraints can disrupt a player's natural rhythm.

### 4.3 Lower Limb, Trunk and Upper Limb Kinematics

Manipulating the timing of tossing arm adduction had multiple ramifications on the full‐body kinematics of the service action. As observed in Table 3, the longer the tossing arm remained up, the more extended the wrist position became at *impact*. Interestingly, wrist angles remained consistent throughout 90%–95% of players' service actions when comparing all three conditions (Figure 3). However, prior to *impact*, players adjusted their wrist extension angle, which affected the angle of the racquet, the penultimate segment of the serve's kinematic chain (Kovacs and Ellenbecker 2011). Consequently, in LATE, with the wrist more extended, the service trajectory became flatter, whereas in EARLY, with the wrist more flexed, the service trajectory became steeper (Table 1). This underscores an important finding for coaches, reaffirming the traditional cues that lowering the tossing arm early results in a steeper service trajectory, whereas the opposite is observed when it remains up for longer.

Recent research has overlooked the kinematics of the head in tennis stroke production, largely owing to measurement challenges (Mayer et al. 2021). This study hypothesised that the head's extension angle at *impact* would be reduced in EARLY, contributing to the steeper downward service trajectory. Surprisingly, there was no significant difference in this variable across the three conditions (Figure 1). The consistency in the head angle between conditions highlights its importance in maintaining eye contact with the ball. Giblin et al. (2017) noted the crucial nature of gaze in the successful completion of the serve and found that when elite‐level players were blindfolded, 16 out of 24 serves failed to contact the ball. Players likely maintain a consistent head position to ensure that the eyes can maintain a ‘fixation point’ with the ball all the way through to *impact* (Lafont 2008). This observation is contrary to the common perception in practice that the early adduction of the tossing arm and flexing of the head are linked. Consequently, for coaches, it is encouraging that the traditional cues influencing the tossing arm do not disrupt head position consistency during the serve.

### 4.4 Limitations

The main limitations of this study included (i) the sample size, (ii) the instructional set provided to participants, (iii) limited analysis of other kinematic parameters related to the tossing arm and (iv) the method used for analysing head position. While the study used a within‐subjects comparison of 10 high‐performance players, a larger sample size would have allowed testing for the potential effects of sex and serve types (foot‐up vs. foot back). For each participant, only the most extreme trials in the EARLY and LATE conditions were selected for analysis. This approach captured a representative sample of the kinematic effects when players interpreted the instructional cues most literally. However, analysing all 30 serves per participant would provide a more balanced representation of the impact of the instructional set. The instructions mirrored traditional coaching delivery, letting players interpret cues in their own way to maintain ecological validity. Nonetheless, measuring the timing of tossing arm adduction for each player would help clarify the relationship between cue interpretation and the resulting kinematic and performance changes.

High‐level tennis players have developed a highly repeatable service motion that is difficult to alter, even after a familiarisation session. As a result, the percentage of successful serves and other kinematic differences may not be solely due to the cue provided but also the difficulty of modifying a well‐rehearsed motor programme. While investigating the timing of tossing arm adduction provided valuable insights, incorporating other parameters—such as the amplitude of adduction, arm trajectory during the upward phase of the toss and its influence on trunk kinematics—would provide a more comprehensive analysis of the tossing arm's kinematics and its effect on the service action.

Statistical parametric mapping analyses would have provided a clearer understanding of the timing and magnitude of kinematic differences across conditions, surpassing the oversimplification of discrete biomechanical parameter analyses. By offering continuous measurement, such analyses allow for a more nuanced qualitative interpretation of the service motion. Finally, the analysis of head position was unable to capture the direction of player gaze, which was only inferred.

### 4.5 Practical Applications

Limited evidence supports traditional coaching cues used in the tennis serve, many of which have evolved through trial and error. The distortion of the serve's rhythm, ball toss and accuracy highlights the need to caution coaches and players about the possible adverse effects of such cues on performance. It also reinforces the importance of validating coaching cues in tennis and other sports to ensure they do not inadvertently impact essential components of skill execution or increase injury risk. Whereas this study confirmed the importance of head position and gaze, further research into their kinematics in tennis and other interceptive sports is encouraged.

## 5 Conclusion

Establishing the theoretical basis behind traditional cues, such as altering the timing of adducting the tossing arm, was a significant gap in the tennis serve literature. This study demonstrated that altering the timing of tossing arm adduction significantly impacted post‐*impact* ball trajectory. When the arm was brought down early, the trajectory became steeper downwards, and when brought down late, the trajectory became flatter, reaffirming common coaching views. While head position remained unchanged between conditions, several other kinematic and spatiotemporal components of the serve were perturbed. Therefore, when providing cues related to the tossing arm, coaches must consider all possible repercussions on the service action, despite achieving the desired effect on post‐impact ball trajectory.

## Conflicts of Interest

The authors declare no conflicts of interest.
